# An Internal Hernia With Small Bowel Volvulus From an Inferior Vena Cava Filter Wire Strut: A Rare Cause of Acute Abdomen

**DOI:** 10.7759/cureus.45499

**Published:** 2023-09-18

**Authors:** Anna Sarkisova, James Nangeroni

**Affiliations:** 1 General Surgery, Rowan-Virtua School of Osteopathic Medicine, Stratford, USA; 2 General Surgery, Southern Ocean Medical Center - Hackensack Meridian Health, Stafford, USA

**Keywords:** small bowel mesentery, inferior vena cava filter retrieval, strangulated internal hernia, closed loop obstruction, mesenteric internal hernia, small bowel ischemia, ivc filter complication, ivc filter migration, mesentery root, small bowel volvulus

## Abstract

The use of inferior vena cava (IVC) filters has been increasingly prevalent. Although they are relatively safe with clear indications, they are not without complications. Late-onset complications include IVC filter migration, IVC wire fracture, wire strut penetration, and perforation of organs in its vicinity. In this report, we present the case of a patient with small bowel volvulus secondary to an IVC wire strut migration, causing tethering of the mesentery and vascular compromise to the small bowel.

## Introduction

The use of inferior vena cava (IVC) filters to prevent pulmonary embolism and deep vein thromboses has been popularized since the 1970s and 1980s [1-2]. With their advent and increased utility, the incidence of pulmonary embolisms has decreased. Even though IVC filters have been shown to be an effective preventative measure, these filters come with a variety of complications such as transmural erosions and migration into nearby and distal anatomic structures [1-2]. The risk of erosion or perforation of an IVC filter is estimated to be as much as 25%, although many patients may be asymptomatic [3]. We report here a unique presentation, evaluation, and treatment of a patient with an IVC penetration by an IVC filter strut and its protrusion into the peritoneal cavity, resulting in small bowel mesentery volvulus around the strut wire and vascular compromise to the small bowel.

## Case presentation

**Figure 1:**
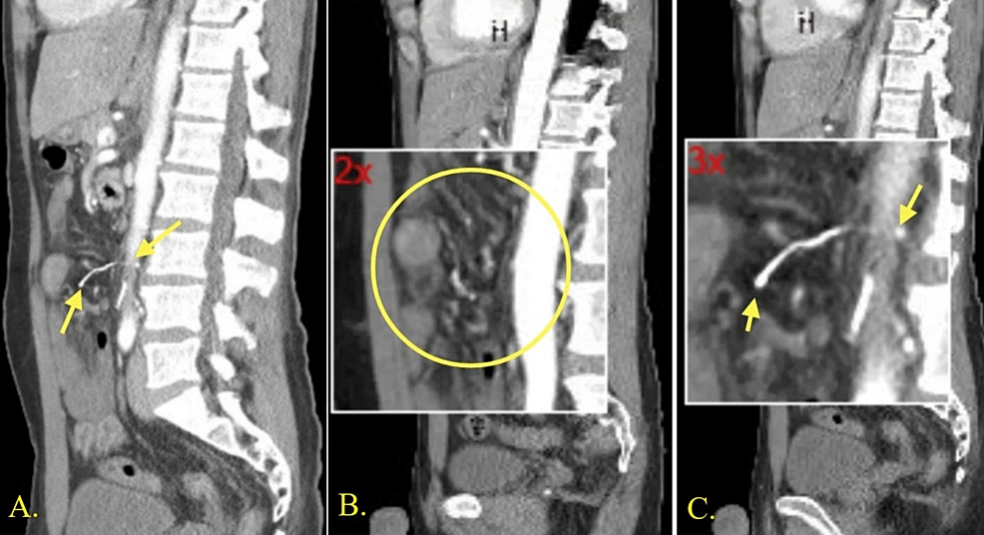
Sagittal views of CT images Images 1A-1C show an IVC filter wire strut measuring 25 mm penetrating the inferior vena cava into the peritoneal cavity CT, computed tomography; IVC, inferior vena cava

**Figure 2:**
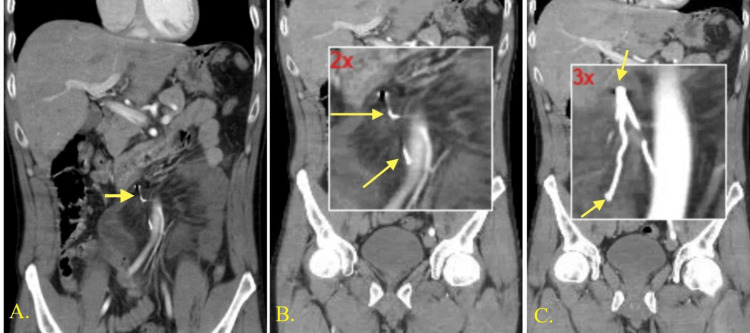
Coronal views of CT images of the abdomen with IV contrast Images 2A-2C show an IVC filter wire strut measuring 25 mm penetrating the inferior vena cava into the peritoneal cavity. Image 2A shows bowel wall changes surrounding the swirl of mesentery and small bowel volvulus. Image 2B shows a piece of IVC filter wire broken off and in the mesentery of the small bowel. Image 2C shows magnification of the wire strut. CT, computed tomography; IV, intravenous; IVC, inferior vena cava

**Figure 3:**
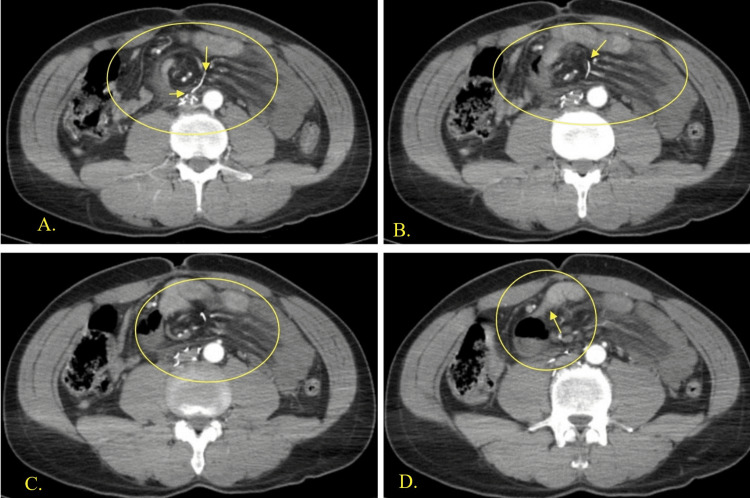
Axial views of CT images of the abdomen with IV contrast Images 3A-3D show an IVC filter wire strut measuring 25 mm penetrating the inferior vena cava into the peritoneal cavity, with progression of mesenteric swirl. Image 3A shows the beginning of wire strut penetration through inferior vena cava. Image 3B shows a piece of IVC filter wire broken off and in the mesentery of the small bowel, creating a mesentery swirl with tunneling. Image 3C shows progression the swirl with bowel wall changes. Image 3D shows the transition point in the obstruction CT, computed tomography; IV, intravenous; IVC, inferior vena cava

We present a case of a patient in his mid-40s with a past medical history of placement of an IVC filter in the mid-1990s after a motor vehicle crash; he was lost to follow-up and without prior history of any other surgeries. This patient presented to the emergency department with multiple episodes of gradually progressing diffuse abdominal pain, nausea, and vomiting of several weeks’ duration. The patient's medical history was significant for placement of an IVC filter in the mid-1990s after a motor vehicle crash. He had no history of prior abdominal surgery and was lost to follow-up many years ago after placement of the IVC filter. The patient ascribed pain as shooting and stabbing, which was exacerbated with movement and relieved by remaining still. He reported having a similar uncomplicated episode of pain that resolved spontaneously at home few weeks prior to arriving to the Emergency Department. Upon physical examination, there was abdominal tenderness on palpation with guarding and signs of peritonitis. The patient was afebrile and hypertensive into 140s/60s with regular heart rate and regular respirations, and his oxygen saturation was above 95% on room air. Given the significance of the physical examination, the patient was urgently taken for radiological evaluation of the abdomen with computed tomography (CT). CT scan with intravenous contrast of the abdomen and pelvis demonstrated small bowel volvulus with possible internal hernia (Figures 1-3). The root of the mesentery appeared to be twisted and edematous, and a positive swirl sign was noted (Figures 3A-3D). Further evaluation of CT images demonstrated that one of the IVC filter wire struts penetrated the IVC, migrated through the retroperitoneum and into the abdominal cavity, and appeared to hook into the small bowel mesentery (Figures 1-3). There was limited small bowel observation secondary to lack of luminal air or contrast material. Laboratory results were mostly unremarkable, except for mild elevation in white blood cell count (11.3×10^9^/L). The rest of the labs were nonremarkable.

Given the patient’s small bowel volvulus findings on CT images with swirl sign and physical examination findings of peritonitis, the patient was taken emergently to the operating room for open abdominal exploration. Intra-operatively, an internal hernia created by mesenteric twisting from IVC wire strut was identified with the jejunal portion of the small bowel looping within its mesentery (Figures 3A-3D). Extensive adhesions were noted throughout the abdominal cavity. A lysis of adhesion was performed with finger blunt dissection. However, there was one area of the mesentery that was severely adhered to the retroperitoneum. Upon further exploration, a steel wire was found extending from the retroperitoneum and pulling onto the small bowel mesentery, which created a tunnel, which resulted in an internal hernia defect. These findings are observed on CT images, which show a piece of IVC filter strut extending out into the peritoneal cavity and hooking into the mesentery, creating a tunneling effect (Figure 3B). A segment of the small bowel was found in the tunnel, creating a closed-loop obstruction with dusky appearance (Figures 2A, 3B, 3D). The mesentery from the wire was freed, and a hemostat was placed around the strut to avoid loss. The mesentery was released, relieving the tethered point, and the small bowel became pink and grossly normal. Next step of the procedure was careful wire strut extraction. To avoid any injury, the wire was exposed as much as possible without penetrating through the retroperitoneum, and the wire strut was cut at the lowest point such that there was no sharp end floating freely in the abdomen. There was no bleeding from the area of the wire, and there was no exposed wire. All of the small bowel was carefully inspected from the ligament of Treitz to the terminal ileum, and all bowel was viable. No bowel resection was performed. The post-operative course was unremarkable. The patient recovered well and was discharged on post-operative day 4. Vascular surgery did not recommend any acute intervention at the time since the IVC strut most likely has been migrating for a very long time and created scar tract. The patient did not follow up with vascular surgery outpatient; however, he did follow up with a primary surgeon a week after his discharge, and the patient's recovery was satisfactory. Pathology report of the extracted IVC filter wire strut identified its length as 25 mm.

## Discussion

Placement of vena cava filters is a surgical intervention performed to prevent pulmonary embolism and deep vein thrombosis, specifically in certain circumstances where medical anticoagulation is contraindicated [4-5]. Although these filters can be implanted in several places, most commonly they are placed in the IVC and less commonly in the superior vena cava [4-5]. Vena cava filters are generally categorized into permanent and retrievable, with permanent filters placed for long-term anticoagulation without a plan to retrieve them. Retrievable filters are placed for short-term anticoagulation with a plan to retrieve the filter at some point in the future [4-5].

Many late-onset complications of IVC filters are uncommon and are reported in individual case reports [3,6-16]. These complications include filter migration, erosion, embolization, and wire fracture [1,17]. Migration of the filter or its components has the capacity to injure neighboring anatomical structures and distal sites. Several cases report damage to the duodenum, pancreas, ureter, abdominal aorta, and bowel [7,9-13,16-17]. In one recent report, a wire strut from an IVC filter migrated through the duodenum, leading to duodenal perforation [3]. Another case report found an extracaval filter strut impinging on the right kidney, duodenum, and colon [13].

To date, literature review shows one case report from 1996, demonstrating a similar case of an IVC filter strut causing small bowel volvulus [6]. One possible mechanism suggested for volvulus to occur is through creation of a fulcrum upon which the mesentery can twist. Prolonged twisting of the mesentery has one ominous outcome with several pathophysiologic mechanisms. The mesentery itself can create a tunneling effect in which a segment of the bowel can become obstructed. The twisting motion of the mesentery can cause a closed-loop bowel obstruction and perforation. Acutely, twisted mesentery can occlude blood flow to the bowel and cause prompt infarction. Even without bowel obstruction, distorted mesentery can lower the perfusion state and create demand ischemia in the bowel and infarction [6].

There are no conclusive study results that support significant safety, efficacy, and complications differences among various types of vena cava filters [18]. The PRESERVE (Predicting the Safety and Effectiveness of Inferior Vena Cava Filters) trial in conjunction with the Society for Vascular Surgery, Society of Interventional Radiology, and U.S. Food and Drug Administration (FDA) initiated a prospective study in 2014 to examine the efficacy, safety, and pattern of IVC filter utility [18]. This study is still ongoing and includes seven IVC filter manufacturers [18]. A review of data from the FDA’s Manufacturer and User Facility Device Experience (MAUDE) database demonstrated that self-reported complication rate with retrievable IVC filters was significantly higher compared with permanent IVC filters [5]. In one observational study of 109 patients, there were two serious procedure-related complications (access site thrombosis and ventricular perforation) [19]. In this study, there were a total of 54 (49.5%) deaths, with a 30-day mortality of 8.3%, and none of them was related to device or procedure [19].

Regarding the patient in our case report, the specific type of filter he had was not known. The permanent IVC filters have been around since the 1980s, and the first retrievable filter was approved by the FDA in 2003 [20]. According to these data and the fact that the patient in this report had the filter placed in the 1990s, it is likely that the IVC filter was a permanent one.

## Conclusions

Even though the overall incidence of very late-onset complications from IVC filters are uncommon, complications from IVC migration pose a great risk of injury of nearby and distal anatomical structures and can lead to devastating clinical outcomes, especially considering that placement of an IVC filter is done for prophylactic indications. Current IVC filters are designed to be explanted at some time in the future. However, there remains an unknown number of people with IVC filters that were lost to follow-up. The clinical significance of this case report outlines the importance of adequate long-term follow-up of patients after IVC filter placement, consideration of repeat imaging, and further consensus guidance on the management of permanent IVC filters.

## References

[1] Hudali T, Zayed A, Karnath B (2015). A fractured inferior vena cava filter strut migrating to the left pulmonary artery. Respir Med Case Rep.

[2] Becker DM, Philbrick JT, Selby JB (1992). Inferior vena cava filters. Indications, safety, effectiveness. Arch Intern Med.

[3] Feezor RJ, Huber TS, Welborn MB 3rd, Schell SR (2002). Duodenal perforation with an inferior vena cava filter: an unusual cause of abdominal pain. J Vasc Surg.

[4] Ghatan CE, Ryu RK (2016). Permanent versus retrievable inferior vena cava filters: rethinking the "one-filter-for-all" approach to mechanical thromboembolic prophylaxis. Semin Intervent Radiol.

[5] Andreoli JM, Lewandowski RJ, Vogelzang RL, Ryu RK (2014). Comparison of complication rates associated with permanent and retrievable inferior vena cava filters: a review of the MAUDE database. J Vasc Interv Radiol.

[6] Lok SY, Adkins J, Asch M (1996). Caval perforation by a Greenfield filter resulting in small-bowel volvulus. J Vasc Interv Radiol.

[7] Lee J, Roche-Nagle G (2021). Permanent IVC filter strut penetration into an abdominal aortic aneurysm. BMJ Case Rep.

[8] Kupferschmid JP, Dickson CS, Townsend RN, Diamond DL (1992). Small-bowel obstruction from an extruded Greenfield filter strut: an unusual late complication. J Vasc Surg.

[9] Khan W, Zhang W, Weiner B, Clark V (2019). 2562 duodenal perforation from an inferior vena cava filter as a rare cause of persistent abdominal pain. Am J Gastroentrol.

[10] Joels CS, Sing RF, Heniford BT (2003). Complications of inferior vena cava filters. Am Surg.

[11] James KV, Sobolewski AP, Lohr JM, Welling RE (1996). Tricuspid insufficiency after intracardiac migration of a Greenfield filter: case report and review of the literature. J Vasc Surg.

[12] Dat A, McCann A, Quinn J, Yeung S (2014). Duodenal perforation by an inferior vena cava filter in a polyarteritis nodosa sufferer. Int J Surg Case Rep.

[13] Goldman KA, Adelman MA (1994). Retroperitoneal caval filter as a source of abdominal pain. Cardiovasc Surg.

[14] Dabbagh A, Chakfé N, Kretz JG (1995). Late complication of a Greenfield filter associating caudal migration and perforation of the abdominal aorta by a ruptured strut. J Vasc Surg.

[15] Marron RM, Rali P, Hountras P, Bull TM (2020). Inferior vena cava filters: past, present, and future. Chest.

[16] Abdel-Aal AK, Ezzeldin IB, Moustafa AS, Ertel N, Oser R (2015). Inferior vena cava filter penetration following Whipple surgical procedure causing ureteral injury. J Radiol Case Rep.

[17] Streiff MB (2000). Vena caval filters: a comprehensive review. Blood.

[18] Gillespie DL, Spies JB, Siami FS, Rectenwald JE, White RA, Johnson MS (2020). Predicting the safety and effectiveness of inferior vena cava filters study: design of a unique safety and effectiveness study of inferior vena cava filters in clinical practice. J Vasc Surg Venous Lymphat Disord.

[19] Chow FC, Chan YC, Cheung GC, Cheng SW (2015). Mid- and long-term outcome of patients with permanent inferior vena cava filters: a single center review. Ann Vasc Surg.

[20] Kaufman JA, Kinney TB, Streiff MB (2006). Guidelines for the use of retrievable and convertible vena cava filters: report from the Society of Interventional Radiology multidisciplinary consensus conference. J Vasc Interv Radiol.

